# Women's higher health risks in the obesogenic environment: a gender nutrition approach to metabolic dimorphism with predictive, preventive, and personalised medicine

**DOI:** 10.1186/1878-5085-4-1

**Published:** 2013-01-12

**Authors:** Niva Shapira

**Affiliations:** 1 Institute for Nutritional Research, Rabin Medical Center (Beilinson Hospital), Office: 5 Kehilat Zitomir, Tel Aviv 69405, Israel

**Keywords:** Women, Gender, Nutrition, Obesity, Metabolic syndrome, Life expectancy, Sexual dimorphism, n-6/n-3 PUFA, Predictive, preventive and personalised medicine (PPPM)

## Abstract

Women's evolution for nurturing and fat accumulation, which historically yielded health and longevity advantages against scarcity, may now be counteracted by increasing risks in the obesogenic environment, recently shown by narrowing gender health gap. Women's differential metabolism/disease risks, i.e. in fat accumulation/distribution, exemplified during puberty/adolescence, suggest gender dimorphism with obesity outcomes. Women's higher body fat percentage than men, even with equal body mass index, may be a better risk predictor. Differential metabolic responses to weight-reduction diets, with women's lower abdominal fat loss, better response to high-protein vs. high-carbohydrate diets, higher risks with sedentariness vs. exercise benefits, and tendency toward delayed manifestation of central obesity, metabolic syndrome, diabetes, cardiovascular disease, and certain cancers until menopause—but accelerated thereafter—suggest a need for differing metabolic and chronological perspectives for prevention/intervention. These perspectives, including women's differential responses to lifestyle changes, strongly support further research with a gender nutrition emphasis within predictive, preventive, and personalized medicine.

## Review

### Introduction

Women's evolution vs. food scarcity, which necessitated effective fat accumulation for preparing available energy and nutrients for fertility and feeding/caring of offspring, has long translated to a health and longevity advantage. However, this may now be counteracted by the ‘obesogenic’ environment.

**Figure 1:**
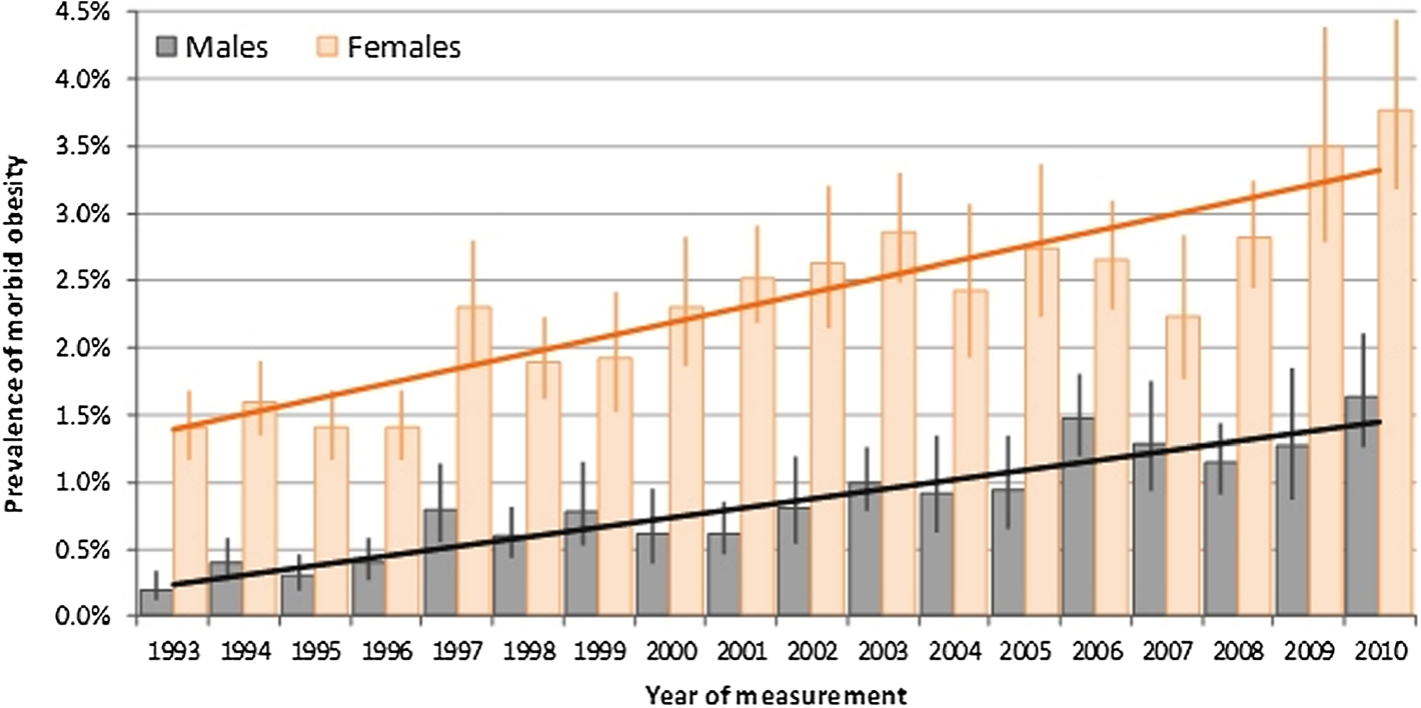
**Prevalence of morbid obesity among adults aged 16+ years: Health Survey for England 1993–2010 **[13]**.** The rise of morbid obesity (≥40 kg/m^2^) has been led by women in developed, high-income countries, i.e. in the UK where between 1993 and 2010, the prevalence of morbid obesity was consistently higher among women (increasing from 1.5% in 1993 to 3.8% in 2010) than among men (increasing from 0.3% in 1993 to 1.6% in 2010).

**Figure 2:**
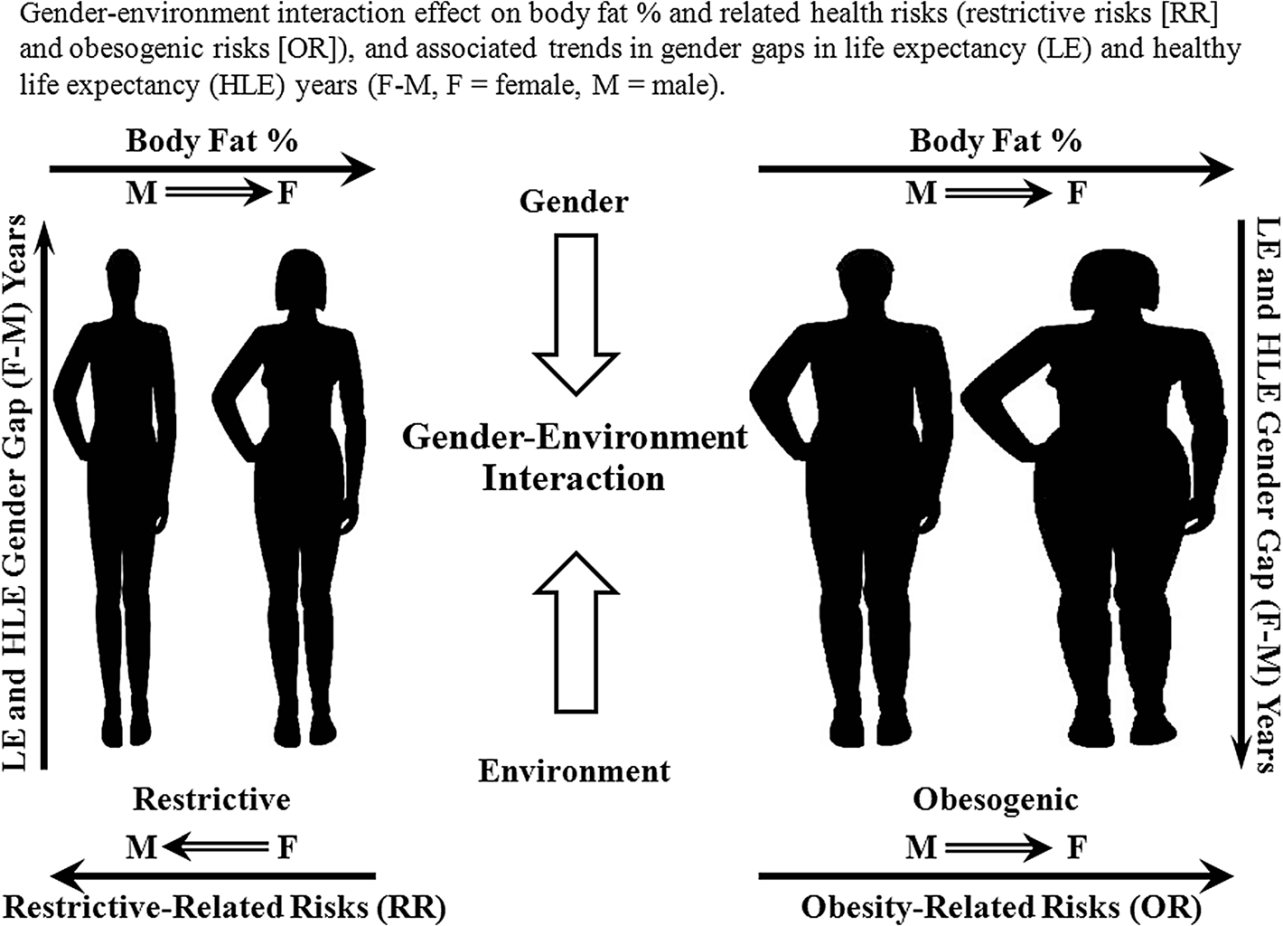
**Gender-environment interaction effect on obesity, health risks, life expectancy (LE), and healthy life expectancy (HLE).** Obesogenic (increased calories and reduced mobility) conditions in Western lifestyles, compared to historically restrictive dietary conditions and high mobility, have conferred a great burden of overconsumption and obesity. Women's innate tendency toward fat accumulation and higher lifelong body fat percentage could make them more vulnerable and have contributed to the recent decline in the gender gap (females-males, years) of life expectancy (LE) and healthy LE (HLE) years.

Pubertal gender dichotomy of girls accumulating fat vs. boys losing fat and growing muscles and height [1,2] illustrates an obesity-related aspect of gender differential adaptation to scarcity and women's advantage. However, extreme changes in the environment, particularly increasing food availability/accessibility and reduced mobility, as well as increased calories and reduced nutrient density in processed foods—together defined as an obesogenic environment—have conferred a great burden of overconsumption and obesity (Figure 1), unrelated to nutritional sufficiency/deficiency [3]. This may be especially critical in females, given their innate tendency toward fat accumulation and risks from nutrient-exhausting pregnancy/lactation, and resultant deficiency disorders [4-6]. This new metabolic ‘mismatch’ in women could greatly contribute to the recent decline in the gender gap in life expectancy (LE) and in healthy LE (HLE), associated with a slowed increase in female LE and HLE compared to males [7-10] (Figure 2), that is gradually narrowing the gender gap [11,12] resulting from increases in the environmental burden on women's health and consequently on the healthcare system.

Beyond the general perspective that the declining gender LE gap was associated primarily with changes in smoking and alcohol use [14], a new perspective on women's health should include their specific metabolic risks and differing timetables, i.e. earlier and higher risk of lifelong obesity, differential fat distribution and risk measures, i.e. body mass index (BMI), waist circumference (WC), and delayed risk manifestation to postmenopausal age, which is associated with reduced estrogen protection. Here, predictive, preventive, personalised nutrition should take a lead—before the risk manifestation—according to the specific timing of physiological events, critical periods, early programming, and their metabolic patterns [15].

The present paper shows women's leading role in the obesity epidemic, which could potentially become their leading lifelong risk factor for disability and mortality. As fewer metabolic studies regarding disease risk have previously been conducted in females, there is a great need for better understanding of women's specific nutritional risks resulting from environmental changes. Approaching metabolic dimorphism as a major factor in gender nutrition is now becoming crucial for designing and enhancing healthcare quality and effectiveness of personalised medicine [16], as comprehensively described in the recent White Paper of the European Association for Predictive, Preventive and Personalised Medicine [17].

### Women's declining advantage in health and life expectancy

Though women still outlive men throughout the world, their LE advantage seen in the early twentieth century is now declining, especially in Western countries [11,12]. For example, in France, previously a gender gap leader (7.66 years), the LE gap stopped increasing in the 1980s and began decreasing in recent years, which was partially attributed to a reduction in cardiovascular disease (CVD) and lung cancer mortality in men, as found in a few European countries [18]. In Norway during the last 25 years, the LE increased by ≈6 years in men and only ≈3 years in women, resulting in a 2.5-year reduction in gender LE gap [7]. In the UK, between 1990 and 2002, the average annual rate of improvement in mortality was ≈30% higher in men than in women [8]. Similar trends had previously been observed in the USA, Sweden, England and Wales, Hungary, Sweden, Australia [19], and Canada [20], though not seen in Japan [21].

Whereas healthy LE has declined in women more than in men, i.e. between 1989 and 2000 by 4.3 years vs. 0.8 years, respectively [18], their unhealthy LE has increased, i.e. women with heart disease have greater LE at 50 years than men, 7.9 vs. 6.7 years, though women's heart disease onset tends to be delayed by ≈3.0 years and heart attacks by 4.4 years compared to men [22]. Similarly, HLE in Italian women was reduced by 2 years compared to their previous advantage, with resultant equal LE at age 65 of ≈7 years, for both genders [9].

A gender health-survival paradox of women's higher morbidity rates despite longer LE—as found in Western countries—is also found in Singapore, where at age 65, women's remaining life yields more disabilities, such as hypertension, bone/joint problems, walking difficulties, and visual and functional impairments compared to same-aged men [10]. The above and further population studies show that women's HLE is compromised beyond their declining longevity, which may necessitate specific preventive strategies.

#### Obesity-related decreased life expectancy and increased disability

Many studies have demonstrated that obese individuals suffer an elevated risk of death [23] and that a high level of obesity is contributing to reduced LE, i.e. in the USA, LE is falling below that of most other industrialised countries, with a ranking of 32nd in the world in 2008 [24], concurrent with the highest per capita expenditure on healthcare in the world [25]. Obesity was associated with reduced US LE at age 50 years by 1.54 and 1.85 years for women and men, respectively, a shortfall (by 42% and 67%) relative to countries with higher LEs, and a higher (by 25% and 40%) effect on LE than in Canada and the UK, the two countries with the next-highest rates of obesity [26]. Excess US BMI was responsible for approximately 95 million years of life lost (YLL), with women accounting for more than two-thirds [27], and their obesity-associated reduction in longevity was higher than that in other countries [28], possibly because of younger age and higher severity of obesity. Of note, an increase in two BMI units in overweight populations was estimated to decrease lifespan, i.e. in men by 1 year, comparable to a 10% increase in the prevalence of smoking [27,29].

**Figure 3:**
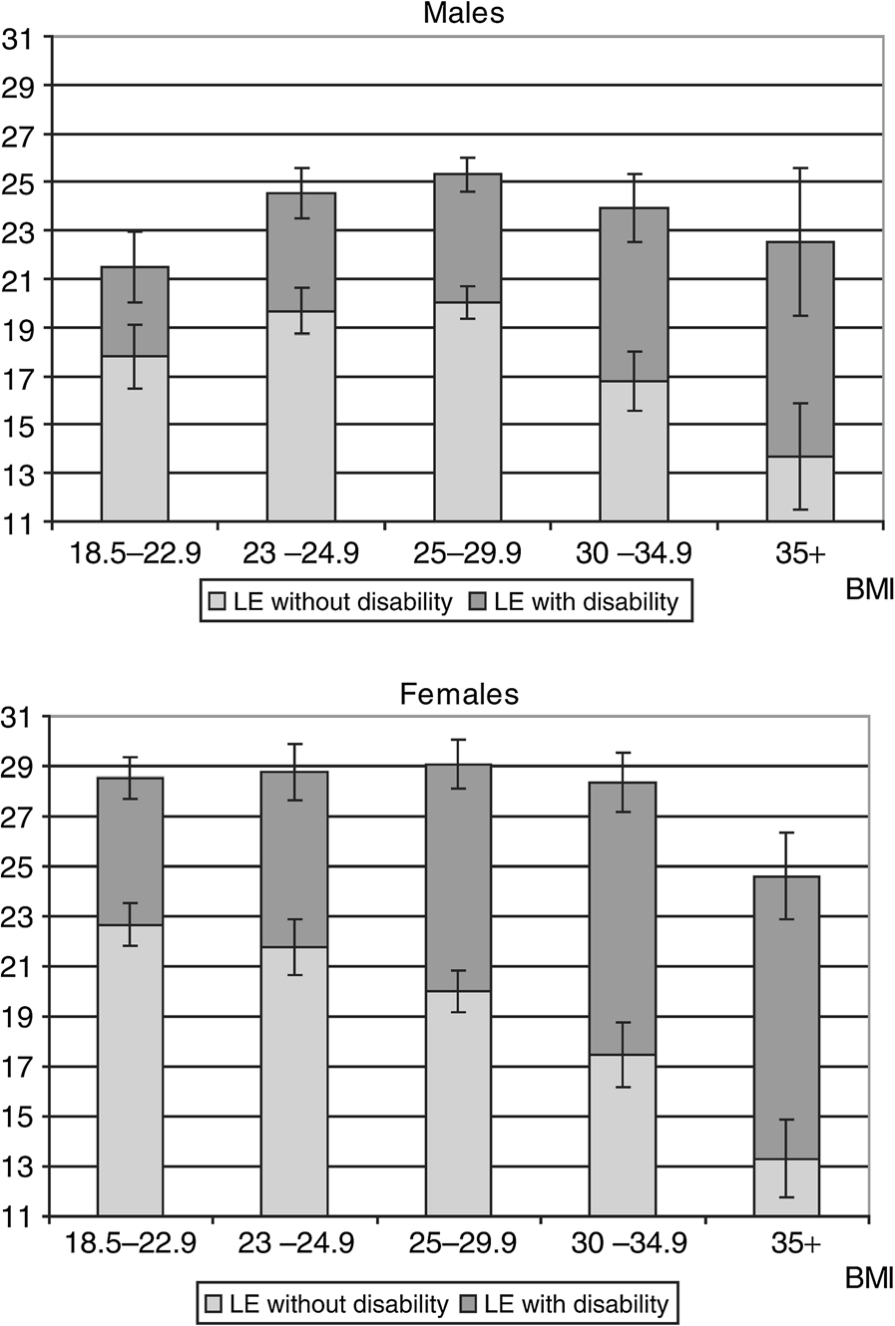
**Life expectancy at age 55 with/without disability in activities of daily living (univariate analysis). ***Error bars* represent 95% confidence intervals of disability-free and total life expectancy [31]. The average LE at 55 years of age is 24.0 years for men and 28.2 years for women (excluding underweight individuals). The longest disability-free LE was found with a BMI between 18.5 and 22.9 for women and 25.0 and 29.9 for men. Mild obesity (BMI 30–34.9) did not shorten total LE, but at age 55, it shortened disability-free life to 2.9 years for males and 4.3 years for females compared to high normal weight (BMI 23–24.9). Severely obese men live an average of 6.0 years less free from ADL disability and women for 8.4 less years. For men, low normal weight (BMI 18.5-22.9) lowers both total and disability-free LE.

Beyond the effect of obesity (BMI of 30–34.9 kg/m2) on reducing LE, it was also shown to reduce disability-free LE [30], i.e. among men by 2.7 years, concurrent with increasing LE with disability by 2.0 years, compared to changes among women by 3.6 years and 3.2 years, respectively, and overweight (BMI 25–29.9) increased LE with disability for women only, by 2.1 years. Women's longest HLE was shown at BMI of 18.5–22.9, men's at 25–29.9 (Figure 3) and decreased thereafter, together with increasing LE with disabilities [31].

#### Women's lead in the obesity epidemic

Worldwide obesity has more than doubled since the 1980s, and rates continue to push upward throughout the world. By 2008, an estimated 1.46 billion adults and 170 million children worldwide were overweight (BMI ≥25 kg/m^2^) or obese (BMI ≥30), with higher rates in women, though varying widely by country. For example, an estimated 18% of women in France are obese, in Greece 26%, in Mexico 35%, and in Saudi Arabia 44%; in contrast, the percentage in both Japan and China was 3% [32]. In the USA, with an overall prevalence of 68.3% overweight [28] and 33.9% obesity, women show higher rates than men of severe obesity (BMI ≥35, 17.8% vs. 10.7%) and morbid obesity (BMI ≥40, 7.2% vs. 4.2%) differences of 78% and 71.4%, respectively [33].

However, longitudinal trends previously showing women's increased obesity prevalence—that initially preceded men's—later slowed, leading to a decline in the gender gap, with women's prevalence over the last 12-year period increasing by only 6.3% vs. men's by 17.1% (1999–2008) [28].

#### Women's body fat percentage vs. BMI as risk-predictive

Though the definition of obesity is uniform for women and men, women typically have higher body fat percentage and lower fat-free mass (FFM) for the same BMI cut-off point [34]. In NHANES III, women's average body fat percentage, at 20–80 years, was higher than men's by 44% (34.9% vs. 24.3%, respectively), despite similar corresponding BMIs (26.27 vs. 26.83). Thus, women's BMI may not accurately reflect but rather may partially mask their actual obesity [34]. This may lead women to a condition of ‘metabolically obese/normal weight’—already at a young age—wherein despite having a normal BMI, they display body composition and metabolic characteristics that may predispose them to development of metabolic syndrome (MetS) [35].

Women's tendency toward obesity compared to men's is manifested by several metabolic patterns, including lower fat oxidation, especially postprandially, with more efficient fat storage [36]; lower resting energy expenditure rates [37-39]; higher response to insulin (as shown in glucose metabolism in both the liver and muscle) and to an exercise with weight loss diet combination [40]; higher adipose tissue-expanding capacity with long-term high-fat feeding [41]; and higher leptin levels, associated with higher inflammatory (C-reactive protein [CRP] and MetS risk, that were independent of adiposity [42].

In metabolically obese normal-weight women, there is a tendency toward greater central fat mass, associated with reduced insulin sensitivity [43,44], shown even with normal glucose tolerance [45]. Further, they may have smaller particles of low-density lipoprotein (LDL); higher concentrations of oxidised LDL, TNF-alpha, interleukin (IL)-6, and leptin; and lower plasma adiponectin than women with normal visceral adiposity [46], all of which contribute to increased obesity-related disease risk. Such hidden obesity was found in underactive Western women and in Asian women, who were observed to have a higher body fat percentage for each BMI level, potentially associated with prominent abdominal obesity, higher intramuscular and liver fat content, and predisposition to insulin resistance and diabetes mellitus [47].

According to body fat percentage, the prevalence of ‘at risk’ (preobese or obese) among normal BMI men and women was 69% and 85%, respectively, suggesting that screening for adiposity in individuals with a normal BMI could further identify those at higher risk for cardiometabolic disturbances and cardiovascular mortality, especially among women, as the false-negative classification of BMI was stronger for women than for men [48]. Together, the above places women at risk for greater obesity and sequelae, especially with increasing exposure to the global obesogenic environment.

#### Women's earlier and greater predisposition to obesity

Women's obesity tendencies begin much earlier than men's, already in the womb [49]. Girls aged ≤10 years have 28% greater total fat and 30% more subcutaneous fat than boys, with similar amounts of visceral fat [1]. Dimorphism in total fat mass and in fat tissue distribution (visceral vs. subcutaneous) progresses from prepuberty [50], where body fat percentage declines in boys as they gain muscle, but increases in girls [2]; correspondingly, early-maturing boys are thinner, whereas early-maturing girls are fatter [51], and menarche seems to occur most frequently with ≥17% body fat [52]. Further, adult women's age of increasing obesity is much earlier than men's, rates higher at 20–39 years of age by 23.7% for BMI ≥30 and 100% for BMI ≥35 and at 40–59 years by 11.4% and 68.1%, respectively [28].

These epidemiological trends and gender differences underscore the importance of defining sex-specific characteristics and women's earlier and stricter prevention and management of obesity and related risks, such as MetS, diabetes mellitus, coronary heart disease (CHD), and cancer [53].

#### Women's delayed risk manifestation: hormonal schedule vs. obesity pressure

Estrogen and estrogen receptors (ER) are well-known regulators of several aspects of metabolism, including glucose and lipid metabolism, and impaired estrogen signalling is associated with the development of metabolic diseases. Here, ERα seems to play a protective role in insulin and glucose metabolism, through effects on the liver, adipose tissue, muscle, and pancreatic β cells and on central regulation of food intake and energy expenditures. ERβ, on the other hand, has the potential to negatively influence insulin and glucose metabolism by impairment of adipose tissue function, probably through augmented PPARγ signalling, and declined expression of GLUT4 in the muscle [54]. Several epidemiological and prospective studies have linked estrogen and the ER to various aspects of metabolic disease and to estrogen protection in premenopausal women.

**Figure 4:**
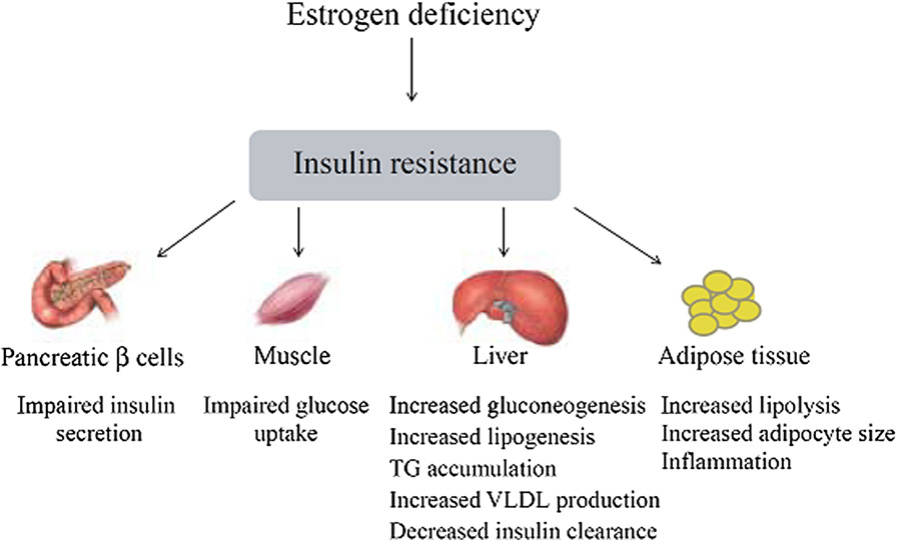
**Overview of insulin resistance induced by estrogen deficiency, and subsequent disturbances in metabolic tissues **[54]**.** As estrogen participates in the regulation of glucose homeostasis, estrogen deficiency, like that seen in post menopausal women, is strongly linked to the development of insulin resistance and subsequent impairments manifested in the pancreas, liver, and muscle and adipose tissue, key organs influencing risk of chronic metabolic disease.

The onset of menopause dramatically increases the risk for women to develop disease states coupled to the MetS, such as obesity, CVD, and type 2 diabetes. Here, estrogen deficiency is strongly linked to the development of insulin resistance and subsequent manifestations in various metabolic tissues (Figure 4) that could be repaired by hormone replacement therapy [54]. For example, estrogen's inverse relation with energy intake, as shown with hormonal shifts during the menstruation/ovulation cycle [52], may partially contribute to young women's ability to control their weight vs. increasing tendency toward obesity with menopause. Similarly, premenopausal women's capacity for removal of very-low-density lipoprotein (VLDL) cholesterol from the plasma is greater compared to men's and to menopausal women's, with resultant rise in the latter's plasma lipoproteins and associated disease risk [55].

However, with increasing obesity levels in young women, MetS and related risks may be manifested earlier in this age group, suggesting that weight gain at an early age predisposes young women to risks as seen with menopausal obesity, due to their earlier and higher rates of obesity-related accumulation of metabolic risks than in men [56].

#### Women-specific chronological perspectives on health risk

Whereas women were previously considered at high CVD risk if their 10-year predicted risk of CHD was more than 20%, their cumulative lifetime risk may be underestimated because of delayed manifestation to postmenopausal age. This may be related to the peak tendency toward weight gain occurring around 50 years of age [57], associated with higher rates of gain [58], difficulty maintaining body weight [59], and tendency toward weight regain following weight loss diets, which is highly predictive of later risk [60]. Updated guidelines from the American Heart Association note that newer risk formulas are available to predict 10- and 30-year risks for all CVD events, including CHD, stroke, and heart failure; for example, the 10-year predicted risk for CHD of >20% now includes women aged >60 years with elevated CRP as candidates for medication intervention, even if they do not have heart disease or elevated lipid levels. Other inflammatory/oxidative medical conditions, such as rheumatoid arthritis, systemic lupus, and a history of pre-eclampsia, gestational diabetes, or pregnancy-induced hypertension, have also been added to the at-risk category. Such additional new high-risk criteria for CVD in women suggest a differential definition of women-specific recommendations for lifestyle and healthcare according to the long-term effectiveness-based paradigm, beyond the evidence-based approach to immediate risk measures [61].

### Women-specific healthy lifestyle aspects

Apart from smoking and/or exposure to environmental (‘second-hand’) tobacco smoke and alcohol, obesity— the suggested leading cause of decreasing gender gap in LE [14] — is associated with most of women's risk factors for MetS and related chronic diseases, i.e. excess body weight/obesity (BMI ≥25), waist circumference (WC ≥35 in. or >88 cm), with elevated blood pressure (≥120/80 mmHg), dyslipidaemia (LDL cholesterol ≥100 mg/dL, high-density lipoprotein (HDL) cholesterol <50 mg/dL, triglycerides (TG) ≥150 mg/dL, and non-HDL cholesterol ≥130 mg/dL) [61], and dysglycaemia [62]. Long-accepted exacerbating factors remain as they were: inadequate physical activity (i.e. <75–150 min/week of moderate-vigorous exercise with an aerobic element) and dietary factors, including low intake of fruits and vegetables, whole-grain/high-fibre foods, and *n*-3 polyunsaturated fatty acid (PUFA; i.e. from *n*-3 PUFA-rich fish^a^ or from supplements), with high intake of saturated fatty acid (SFA), cholesterol, alcohol, sodium, sugar, and *trans*-FA (*t*FA) [61]. Low socioeconomic/cultural status is another risk factor highly associated with increased obesity, especially in women—found to be key in a recent re-evaluation of the United States Mortality File [63]—and in low- and medium-developed countries [64], requiring specific considerations and economic and cultural approaches that are beyond the scope of this manuscript.

Healthy lifestyle in women that was associated with a significantly reduced risk of total and ischaemic stroke [65] consisted of no smoking, low BMI (≤22), moderate alcohol consumption (4–10.5 drinks per week^b^), regular exercise (more than four times per week), and a healthy diet, incorporating high fibre, folate, and *n*-3 PUFA, with generally high PUFA/SFA ratio, and low *t*FA and glycaemic load (GL). Adherence to these lifestyle guidelines could dramatically reduce the risk of CHD by approximately 82% [66]. Additionally, adherence to the American Cancer Society's prevention guidelines—including for BMI, physical activity, diet, and alcohol consumption—lowered the risk of cancer and all-cause mortality in non-smokers [67]. Further detailed recommendations for primary prevention included adjusted intake of meats and fatty foods—especially sources of *n*-6 and *n*-3 PUFA—adding olive oil, selected vegetables, and citrus fruits, and adequate body fat/lean mass proportions [68]. A dietary pattern high in fruit and low-fat dairy and low in white bread, processed meat, margarine, and soft drinks was suggested to help prevent abdominal fat accumulation [69]. The above suggests that major Western diseases share a common metabolic-nutritional basis and thus require similar preventive measures. However, the alcohol link to cancer risk [70-72] vs. benefits to heart health and diabetes [73] is a reminder that the specificity of foods and risk factors should not be overlooked, especially with regard to the gender aspect [70,71,74,75]. The Mediterranean diet that has repeatedly demonstrated an advantage against Western diseases and was further suggested as a nutritional framework for the predictive, preventive, and personalised medicine (PPPM) approach [76] can be effectively adapted and applied to various metabolic states, including those associated with gender-specific risks.

#### Women-specific aspects of weight management

The general combination of reduced calorie diets and exercise (both aerobic and resistance) [77] was repeatedly confirmed in both sexes to be effective in muscle preservation, preferential reduction of abdominal vs. subcutaneous adipose tissue, and improvement in fitness capacity compared to diet alone [78]. However, such a combination, which was further associated with a twofold greater improvement in insulin action compared with diet alone in men [79], did not show similar effectiveness in women [80], in whom exercise alone, even without caloric restriction and/or weight loss, was associated with reduced total and abdominal obesity and insulin resistance [81].

Women's fat loss, primarily subcutaneous vs. intra-abdominal in men, yields much smaller improvements in their specific risk factors, such as TG and HDL cholesterol levels, compared to men losing the same amount of weight/fat, but mostly abdominal [82]. Though WC is known to be positively associated with diabetes in both sexes, increasing WC was more closely associated with diabetes in women than in men [83], which may suggest higher sensitivity of WC as a metabolic measure in women. The above suggests women's need for specific metabolic emphases in obesity management, beyond BMI and weight loss diet *per se*, vs. their specific risks and chronological aspects compared to men's achieving better risk reduction already through weight loss and dietary restriction [84].

#### Dietary macronutrients and metabolic aspects

##### Fats

A low-fat/cholesterol diet is routinely recommended for individuals with elevated plasma LDL cholesterol concentrations [85], though the combination of a weight loss diet with exercise was less effective in improving lipoprotein levels and LDL size in women than in men [86]. A diet low in fat (25% kcal), SFA (7%), and cholesterol (100 mg/day)—consistent with the NCEP Step II diet—only partially attenuated the increase in LDL cholesterol during menopause onset [87]. A diet low in fat and high in vegetables, fruit, and whole grains in the Women's Health Initiative study showed women to have a smaller decrease in plasma lipoprotein levels, similar decreases in particle sizes of LDL and HDL, but greater reductions in postprandial TG levels compared to men [85]. As increasing carbohydrate intake may increase women's risk more than men's [88], a low-fat diet with carbohydrate substitution may not necessarily provide women with the metabolic protection against obesity-related risk that has been shown in men. These findings may suggest a need for gender-based dietary interventions to improve specific risk factors, with awareness of women's differential response to strategies that have successfully been targeted toward men [89].

##### Fatty acids

Both essential fatty acids (EFA), linoleic acid (LA; 18:2 *n*-6) and alpha-linolenic acid (ALA; 18:3 *n*-3), are known for their lipidemic advantages, i.e. for reducing LDL cholesterol and TG. Substitution for SFA with *n*-6 PUFA has demonstrated an advantage in reducing LDL/HDL and TC/HDL ratios and TG levels, thus improving metabolic factors and related effects in both men and women. However, the unsaturated character of PUFA may be associated with greater lipid and LDL oxidation, especially with inflammation, i.e. facilitated by high *n*-6 proinflammatory eicosanoids, believed to play a key role in chronic diseases and accelerated ageing in conditions of a high-*n*-6 Western diet [90,91]. Essential PUFA are further converted in the liver to LCPUFA, LA into arachidonic acid (20:4 *n*-6), and ALA into eicosapentaenoic acid (20:5 *n*-3) and docosahexaenoic acid (22:6 *n*-3). Their conversion varies according to gender and age, being highest in young women—especially during pregnancy [92,93]—compared to males [94,95] and declining with age along with levels of the rate-limiting enzymes delta-5- and delta-6-desaturases, more in women than in men [96]. Desaturase activity is reduced with high SFA intakes [97] and cholesterol [98] and increased with a high-*n*-3 PUFA and/or MUFA diet [97]. LA (*n*-6) and ALA (*n*-3) share and compete for rate-limiting conversion enzymes, emphasizing the importance of the *n*-6/*n*-3 PUFA ratio for health and sex-related differential predisposition. Women's higher production of eicosanoids from either dietary *n*-6 or *n*-3 PUFA, which are pro- and/or anti-inflammation/coagulation/carcinogenesis, respectively, yields stronger implications for differential CVD and cancer pathophysiology and clinical outcomes, corresponding to their dietary PUFA ratios, as compared to men's.

An ‘*n*-6 Gender Paradox’ hypothesis’ was proposed, based on the Israeli case study of women's higher risk with *n*-6 PUFA vs. men's benefit. Here, women's worse health ranking vs. men's relative advantage in conditions of high dietary *n*-6 PUFA (10–12% kcal) [99] led to the unexpected observation of low national health status, previously defined as the ‘Israeli Paradox’ [100,101]. The above suggests that with the same diet, at certain high levels of *n*-6 PUFA, women's greater transformation to eicosanoids with proinflammatory, carcinogenic, aggregatory characteristics may put them at greater risk compared to men, who may benefit due to lower EFA transformative capacity. In a recent Danish epidemiological study, an *n*-6 PUFA increase was associated with weight gain and increased WC in women, while the opposite was shown in men [102], which could further support a differential response and high-*n*-6 risk for women vs. men's advantage.

##### Carbohydrates and glycaemic effects

High carbohydrate intake may be associated with a disadvantage for the lipid profile, including high TG and VLDL, especially in overweight postmenopausal women [103], reflecting the high impact of insulin on their lipid metabolism [38]. Concurrent decreased LDL particle size further explains the link between high dietary carbohydrates and women's CVD risk [104].

While in men replacing SFA with carbohydrate from grains, vegetables, legumes, and fruit effectively reduced total and LDL cholesterol blood levels [87], only a moderate reduction was observed in women, with lesser effects on CVD [89]. Replacement of SFA by carbohydrates, especially refined sources and ‘added sugars’, increases plasma TG and small LDL particles and reduces HDL, which are of particular concern in the context of the increased prevalence of obesity and insulin resistance, all especially critical for women [88].

In the EPICOR study, women in the highest quartiles of carbohydrate intake, GI, and GL had a significantly greater risk of CHD (by about twofold) than those in the lowest quartile, while a lesser association was found in men [88]. A twofold increased CHD risk with high GL, most evident among women with BMI ≥23, was found in follow-up research over a 10-year period (729,472 person-years), but not among normal-weight women [105]. Dietary GI and GL were progressively associated with CHD in various populations [106] and with plasma CRP levels, in general and in healthy middle-aged women [107], suggesting an explanatory link between women's ischaemic heart disease with overweight and their susceptibility to insulin resistance [108].

##### Proteins

Although energy restriction alone often leads to weight loss, the composition of the lost tissue also matters, and high loss of lean mass could have deleterious metabolic consequences. This is especially critical for women with innately low initial FFM, a tendency toward a plateau in weight loss, and for later weight regain [56,109]. Moreover, because skeletal muscles play roles in energy metabolism, their potential loss in the weight reduction process emphasises the need to focus on the composition of the lost weight for preservation of lean tissue, rather than relating merely to scale weight. Here, higher protein, lower carbohydrate, and GI energy-restricted diets have been shown to help offset women's lean mass loss, especially when associated with resistance exercise [110].

A high-protein diet was more effective for women, who lost nearly twofold more total and abdominal fat compared with women on the low-protein diet, whereas in men, there was a lesser difference in fat loss between diets; in both sexes, a high-protein diet caused greater total and LDL cholesterol reductions [111,112], with no effect on blood TG [112]. A high-protein (≥40% kcal) diet was also more effective in women with polycystic ovary syndrome, yielding a greater reduction in body weight, body fat, WC, and blood glucose than the standard protein (15% kcal) diet after 6 months [113]. A high-protein diet (1:1 vs. 3:1 carbohydrate/protein ratios) was superior to a low-fat and high-carbohydrate diet, with or without an aerobic/resistance training programme, for effective weight loss, nitrogen balance, improved body composition, and reduced risk factors for the MetS in overweight and obese women [114]. A high-protein, high-dairy, energy-restricted diet plus exercise combination was further linked to improved energy-protein balance compared to a lower dairy, higher protein diet, as shown by greater losses of total and visceral fat, smaller losses of lean mass, and increases in body strength despite identical weight loss [110]. Moreover, during adolescence, higher dairy product intake was associated with a lower risk of later adult type 2 diabetes, partially explained by the persistence of the consumption pattern through adulthood and results of a cumulative high-protein, high-dairy effect [115]. A high-protein, low-carbohydrate diet was also found to reduce blood glucose, insulin, and lactate levels and to prevent cancer initiation and to slow tumour growth [113].

#### Physical activity

Sedentary life, a characteristic of the modern environment, is a known health risk factor. Moreover, increased sitting time was recently found to be an active and independent risk factor, positively associated with fasting insulin, leptin, leptin/adiponectin ratio, CRP, and IL-6 in women, more than in men. These associations remained significant after additional adjustment for total moderate- to vigorous-intensity physical activity [116]. In contrast, physical activity was inversely correlated with BMI, insulin levels, CRP, leptin, WC, and body fat percentage in young and middle-aged women, suggesting ‘anti-age’-related increases in the above measures with physical activity that may potentially counteract sedentariness and age effects in women [117]. Additionally, non-exercise activity, all activity that is not sleeping, eating, or sports-like exercise, could be a critical component for increasing energy expenditure and metabolic rate, maintaining FFM, and preventing weight gain, obesity, and sedentariness-related risk [81].

As women oxidise proportionately more lipids and less carbohydrates and protein compared to men, and as they do not build muscle glycogen with a carbohydrate load but rather more body fat, their exercise-related fat loss is critical for improved body composition [116,118]. Exercise-associated increases in lipolysis in abdominal visceral fat and reduction of their related risks, despite a lower response from luteal-femoral adipose tissue, explains the higher exercise-related benefit and reduction in women's morbidity and mortality, independent of BMI or weight loss, compared to men [119].

A combined exercise and weight loss diet was associated with the greatest reduction in women's total, abdominal, and subcutaneous fat; reduced insulin resistance (≈32%) in the exercise plus diet group, but not with diet alone; and WC closely reflecting the benefits of reducing abdominal obesity, whereas BMI alone may mask the positive effects of exercise [81]. The correlation between recreational physical activity and reduction in risk of breast cancer recurrence and mortality, which are known to be associated with increased abdominal obesity, further support the special importance of physical activity to women [120].

## Conclusions

Findings showing women's differential metabolic respon-ses have suggested a gender effect on biochemical-endocrinological patterns, metabolic mechanisms, and risk factors, emphasising the importance of more gender-specific prevention strategies. This is especially relevant vs. environmental changes and the obesogenic epidemic, with women's lead in earlier and higher obesity rates and related disease risk, though with manifestation mostly delayed to menopausal age.

Women's differential metabolic responses compared to men throughout the life cycle strongly suggest a need for gender-specific strategies against obesity and chronic diseases such as CVD, diabetes, MetS, and cancer, including differential metabolic biomarkers and chronological patterns across the spectrum of diseases. This is especially relevant in light of women's unique vulnerability to modern environmental pressures, including increased sedentary lifestyle, obesity, glycaemic load, dietary *n*-6/*n*-3 PUFA ratio, and transitional socioeconomic and psychosocial stresses. Applying men's knowledge to women's practice may not only yield lower benefits, but may also further enable exacerbation of metabolic imbalance, i.e. substituting dietary fat with carbohydrates and/or repeated weight loss diets without considering the preservation of women's lean body mass, which may gradually reduce their metabolic balance and resistance to Western diseases, despite greater benefits for men. Further, the differential response to higher protein/carbohydrate ratios, to low glycaemic load, as well as to exercise vs. sedentary lifestyle should be considered for differential prevention and intervention strategies. A differential time perspective is also required, considering females' much earlier fat accumulation process, which presets the metabolic patterns for later increases in obesity risks compared to males. Even a measure considered basic, i.e. BMI, may underestimate the female obesity state and rather masks fat percentage, which could better reflect the metabolic obesity state, which is more closely associated with obesity risk, especially with android type, manifested by abdominal subcutaneous and visceral fat and high WC.

As CVD prevention shares recommendations with those of cancer as well as other chronic Western diseases, women's approach may be better based on their specific metabolic risks, biomarkers, and chronological patterns across the spectrum of conditions, beyond specific diagnosis, prevention, and intervention. Much epidemiological study and clinical research are needed, including interventional trials for attaining women-specific understanding of metabolic risks and epidemiological evidence-based recommendations for designing targeted nutritional strategies within the context of gender nutrition and the PPPM approach to health- care.

## Endnotes

^a^ Pregnant women are generally counselled to avoid eating types of fish with the potential for the highest level of mercury contamination (i.e. shark, swordfish, king mackerel, or tile fish).

^b^ The American Cancer Society's recommendation for alcohol intake was recently reduced to no more than one drink per day for women and two for men [121], in response to findings of alcohol-cancer links [74] even with intake previously considered ‘low’ [61,70,71,74,75].

## Abbreviations

ALA: Alpha-linolenic acid; BMI: Body mass index; CHD: Coronary heart disease; CRP: C-reactive protein; CVD: Cardiovascular disease; EFA: Essential fatty acid; ER: Estrogen receptor; FA: Fatty acid; FFM: Fat-free mass; GI: Glycaemic index; GL: Glycaemic load; HDL: High-density lipoprotein cholesterol; HLE: Healthy life expectancy; LA: Linoleic acid; LCPUFA: Long-chain polyunsaturated fatty acid; LDL: Low-density lipoprotein cholesterol; LE: Life expectancy; MetS: mMetabolic syndrome; MUFA: Monounsaturated fatty acid; *n*-: Omega (-3, -6, -9 unsaturated fatty acids); NCEP: National Cholesterol Education Project; NHANES: National Health and Nutrition Examination Survey; PPPM: Predictive, Preventive, and Personalised Medicine; PUFA: Polyunsaturated fatty acid; SFA: Saturated fatty acid; *t*FA: *Trans*-fatty acid; TG: Triglycerides; TNF: Tumour necrosis factor; VLDL: Very-low-density lipoprotein cholesterol; WC: Waist circumference.

## Competing interests

The author declares that she has no competing interests.
